# 3-D thermal regime and dehydration processes around the regions of slow earthquakes along the Ryukyu Trench

**DOI:** 10.1038/s41598-021-90199-2

**Published:** 2021-05-27

**Authors:** Nobuaki Suenaga, Shoichi Yoshioka, Yingfeng Ji

**Affiliations:** 1 Research Center for Urban Safety and Security, Kobe University, Rokkodai-cho 1-1, Nada ward, Kobe, 657-8501 Japan; 2 Department of Planetology, Graduate School of Science, Kobe University, Rokkodai-cho 1-1, Nada ward, Kobe, 657-8501 Japan; 3 State Key Laboratory of Tibetan Plateau Earth System Science (LATPES), Institute of Tibetan Plateau Research, Chinese Academy of Sciences, Beijing, 100101 China

**Keywords:** Geophysics, Tectonics

## Abstract

Several interplate seismic events, such as short-term slow slip events (S-SSEs) and low-frequency earthquakes (LFEs), have been identified in the Ryukyu Trench, southwestern Japan. As one of the specific characteristics of this seismicity, the depths at which S-SSEs occur at the plate interface beneath Okinawa Island are approximately 5–10 km shallower than those beneath the Yaeyama Islands. To elucidate the cause of this difference in depth, we constructed a three-dimensional, Cartesian thermomechanical subduction model and applied the subduction history of the Philippine Sea (PHS) plate in the model region. As a result, the interplate temperatures at which S-SSEs take place were estimated to range from 350 to 450 °C beneath Okinawa Island and from 500 to 600 °C beneath the Yaeyama Islands. The former temperature range is consistent with previous thermal modelling studies for the occurrence of slow earthquakes, but the latter temperature range is by approximately 150 °C higher than the former. Therefore, explaining how the depth difference in S-SSEs could be caused from the aspect of only the thermal regime is difficult. Using phase diagrams for hydrous minerals in the oceanic crust and mantle wedge, we also estimated the water content distribution on and above the plate interface of the PHS plate. Near the S-SSE fault planes, almost the same amount of dehydration associated with phase transformations of hydrous minerals from blueschist to amphibolite and from amphibolite to amphibole eclogite within the oceanic crust were inferred along Okinawa Island and the Yaeyama Islands, respectively. On the other hand, the phase transformations within the mantle wedge were inferred only beneath the Yaeyama Islands, whereas no specific phase transformation was inferred beneath Okinawa Island around the S-SSE occurrence region. Therefore, we conclude that dehydrated fluid derived from the oceanic crust at the plate interface would play a key role in the occurrence of S-SSEs.

## Introduction

**Figure 1:**
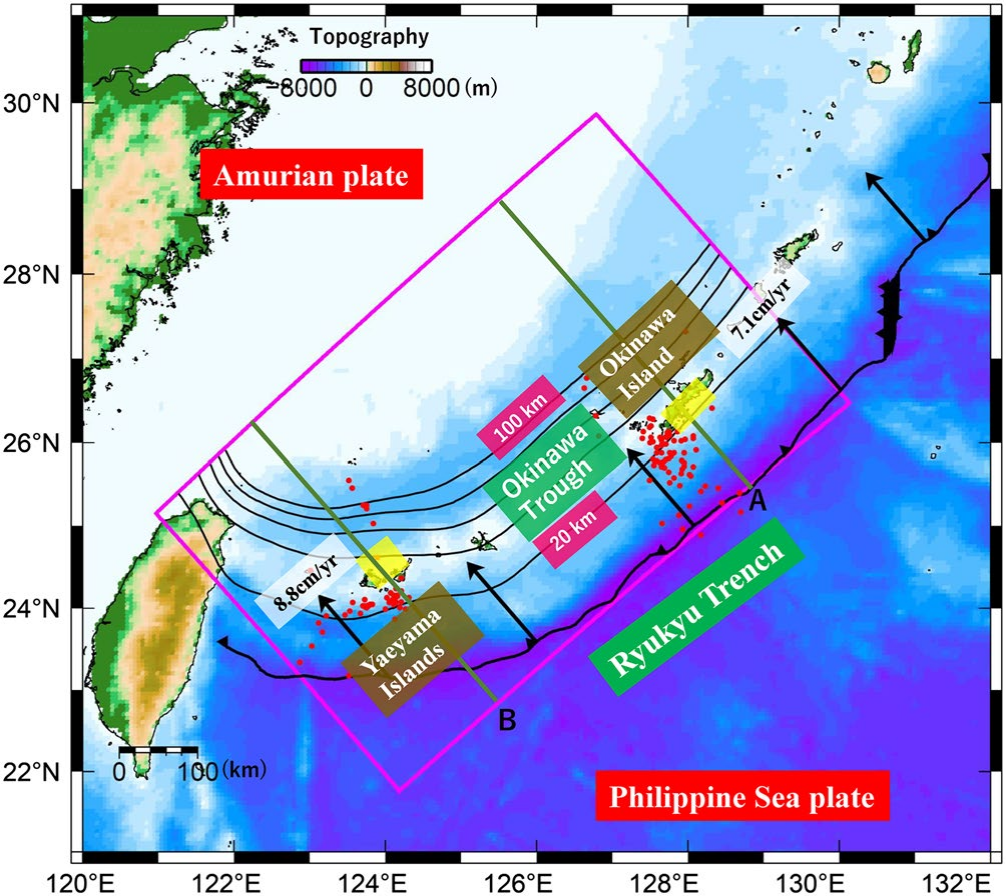
Tectonic map of the Ryukyu subduction zone and its around area, southwestern Japan. The black barbed line represents the Ryukyu Trench. The black arrows denote plate motion vectors of the Philippine Sea (PHS) plate with respect to the Amurian plate^1^. The rectangular region surrounded by the pink lines denotes the model region. The black solid lines with numerals represent isodepth contours of the upper surface of the subducting PHS plate (in km) in the model region^16–17^ whose depth intervals are 20 km. The yellow-shaded rectangular areas denote the fault planes of S-SSEs^2^ during the period from 1 January 1997 to 10 November 2013. The red-solid circles denote epicentres of LFEs^3^ during the period from 1 April 2004 to 31 December 2016.

In southwestern Japan, the Philippine Sea (PHS) plate is subducting beneath the Amurian plate along the Ryukyu Trench^1^ (Fig. 1). As the Global Navigation Satellite System (GNSS) and seismic observation networks have developed in recent years, slow earthquakes, such as short-term slow slip events (S-SSEs)^2^, low-frequency earthquakes (LFEs)^3^, very low-frequency earthquakes (VLFEs)^4^, and tectonic tremors^5^, have been identified along the Ryukyu subduction zone. One of the most notable features of the slow earthquake distributions in and around this area, revealed by recent studies, is that there is a difference in the source depths of S-SSEs beneath the Okinawa Island region and beneath the Yaeyama Islands region^3^. In the Okinawa Island region, S-SSEs take place at the plate interface at a depth of approximately 25 km (Fig. 1). The depth at which these S-SSEs occur is almost the same as that of LFE swarms that occur around this region, although the latter depths were not well constrained. On the other hand, in the Yaeyama Islands region, S-SSEs take place adjacent to the LFE active region at a depth of approximately 35 km, which is 10 km deeper than that beneath the Okinawa Island region. For the LFE distributions, southward migrations have been identified beneath both Okinawa Island and the Yaeyama Islands^3^. This mobility of the source of LFEs would indicate the existence of fluid near the plate boundary^6^. By incorporating a modified network inversion filter into GNSS time series data, the spatiotemporal source processes of five S-SSEs in the Yaeyama Islands region during the period from March 2010 to February 2013 were revealed, and LFE and VLFE activity was inferred to have initiated after the evolution of S-SSEs^7^. Considering the source mobility of LFEs and the synchronization among their occurrences, S-SSEs would activate the LFEs and VLFEs associated with fluid transport near the plate interface. The thermomechanical subduction modelling in the preceding study implied that the transition in the slip mode may be constrained by temperature–pressure conditions at the plate interface^8^. Previous studies discussing the relationship between permeability and fault strength^9–10^ suggested that low permeability in the hanging wall results from serpentinization or deposition of silica due to dehydration from the slab near the mantle wedge corner, which would prompt the formation of a high pore fluid pressure region at the plate interface and reduce the effective normal stress there. Then, episodic tremor and slip (ETS) may occur at an isolated friction zone within this region. Low effective normal stress in the ETS zone resulting from high pore pressure and chemical dehydration reactions around the plate interface may induce semi-brittle deformation of serpentinite, which is governed by both brittle and ductile responses and has a low healing rate^11^. A variation in the slip behaviour from velocity strengthening to velocity weakening has been identified there, which is reasonable for the slip mechanism of slow earthquakes^12^. Another friction model^13^ also supports the presence of the high pore pressure region to reproduce realistic properties of slow slip events. In addition to frictional properties, the plastic flow of antigorite-rich serpentinite influences the dynamics of fault slip^14^.

To investigate the similarity or difference between the Okinawa Island and Yaeyama Islands regions with respect to the thermal regimes and the dehydration processes near the S-SSEs and LFEs, we performed several numerical simulations of temperature fields associated with the subduction of the PHS plate using a 3-D Cartesian thermomechanical subduction model and estimated the water content distributions on and above the subducting PHS plate using phase diagrams for hydrous mid-ocean ridge basalt (MORB) of the oceanic crust and ultramafic rocks of the mantle wedge. We took certain parameters as unknowns and tested their sensitivity to the thermal regime. Then, we determined the most suitable model to fit the calculated thermal regime with the observed heat flow data.

## Model and tectonic setting

**Figure 2:**
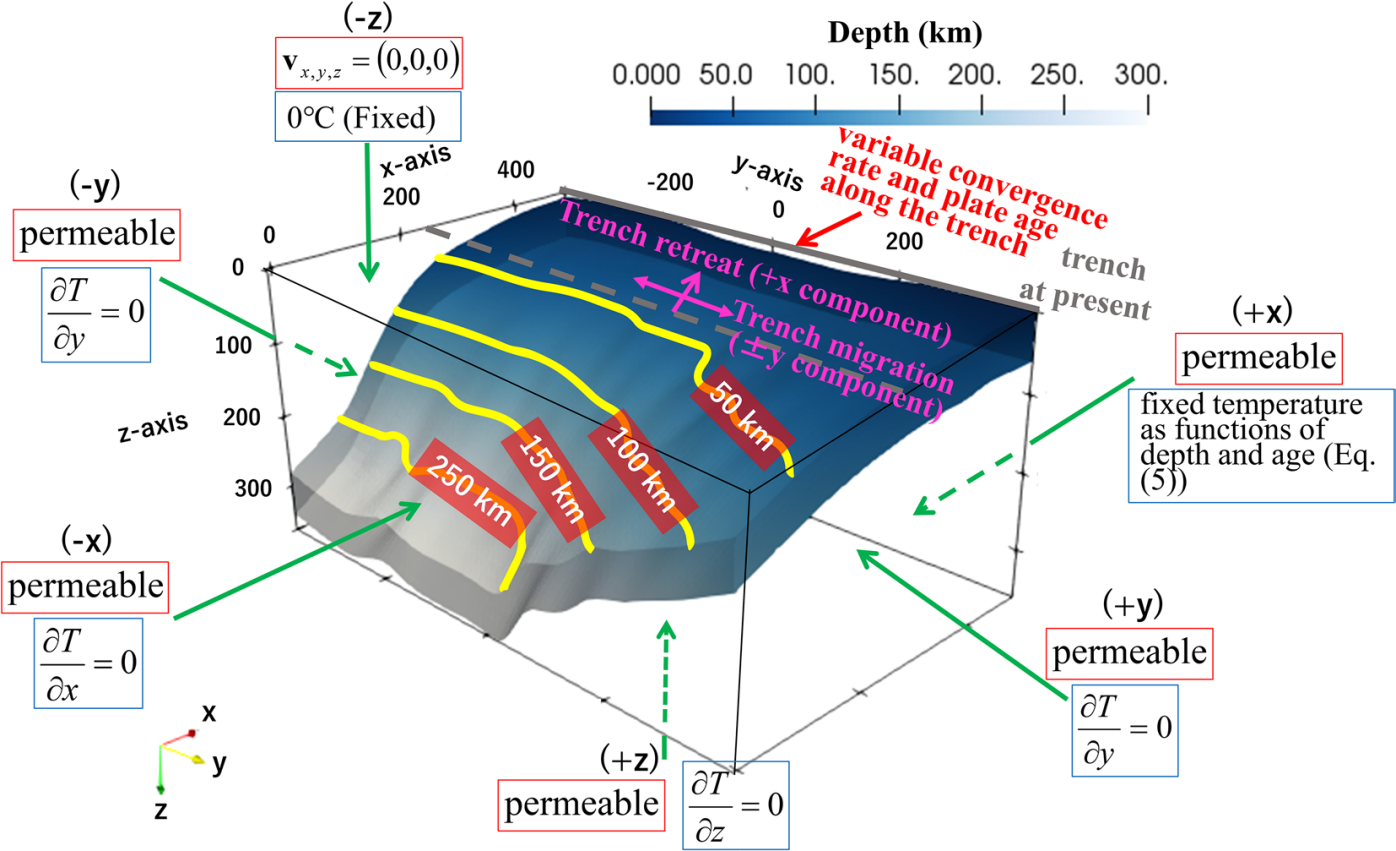
Schematic figure of the 3-D Cartesian thermomechanical subduction model used in this study. The mantle flow and thermal boundary conditions at respective boundaries are noted by red and blue surrounded lines, respectively. The yellow solid lines on the plate interface denote isodepth contours of the subducting PHS plate.

Following Ji and Yoshioka (2015)^15^, we constructed a 3-D Cartesian thermomechanical subduction model and performed numerical simulations during the period from 15 Ma to the present (0 Ma) to calculate the spatiotemporally changing temperature structure and mantle flow velocity field associated with the subduction of the PHS plate. The dimensions of the model were 500 km, 800 km, and 400 km in the x-, y-, and z-axis directions, respectively (Fig. 2). The numbers of nodes used were 50, 80, and 80 in the x, y, and z directions, respectively. The geometry of the subducting PHS plate was created by combining the Slab 2.0 model^16^ with Yamamoto et al. (2018)^17^, which is based on a seismic velocity structure survey around the Yaeyama Islands. The subduction of the PHS plate was modelled kinematically, and the PHS plate was constrained to subduct along a prescribed guide with an imposed convergence rate (Fig. 2). The model consisted of the subducting oceanic plate (within the prescribed guide), continental upper crust, continental lower crust, accretionary prism, and mantle (Fig. S1). The domains of the upper crust, lower crust, and accretionary prism were rigid; the other domains were composed of convective regions.

For the tectonic setting associated with the subduction of the PHS plate along the Ryukyu Trench, we referred to a global plate rotation model^18^ (Fig. S2) and a trench motion model^19^. From 15 to 3 Ma, the PHS plate moved in a northeasterly direction. Subsequently, the PHS plate changed its motion to a northwesterly direction at 3 Ma. In addition, the Ryukyu Trench retreated southeastward^19^; the Okinawa Trough, which is an active back-arc basin of the Ryukyu arc, initiated spreading associated with subduction of the PHS plate at approximately 2 Ma^20–21^. The spreading rate increased towards the southwest^22^, although its exact value from 2 Ma to the present is unclear. In this study, we evaluated the validity of the estimated thermal structures by comparing calculated heat flows with observed heat flows. For heat flow data, we used the Global Heat Flow Database (GHFD)^23^ and land boreholes^24–25^. To compare the observed heat flows with the calculated heat flows, we used the following equation:

1 $$ S = \frac{1}{N}\left[ {\sum\nolimits_{i = 1}^{N} {\left( {{hf^{obs}_i} - {hf^{cal}_i} } \right)^{2} } } \right]^{\frac{1}{2}} $$

where ${hf^{obs}_i}$ and ${hf^{cal}_i}$ are the *i*th observed and calculated heat flow values, respectively, and $N$ is the total number of data points from land boreholes and marine heat probes within the model region. We constructed a best-fit model to minimise $S$ in Eq. (1).

We investigated several parameter combinations of the trench retreat rate and initiation age of the trench retreat, and compared the calculated heat flow values with the observed heat flow values to evaluate the parameter dependency on the trench retreat effect. For the parameter search range, the trench retreat rate was assumed to range from 15 to 20 mm/year^19^, and the initiation age of the trench retreat was set to range from 8 to 5 Ma, following a previous thermal modelling study in this region^26^. Furthermore, we considered the spreading rate of the Okinawa Trough, which ranged from 5 to 20 mm/year^22^ uniformly in the + x direction (Fig. 2) from 2 Ma to the present, including the initiation of rifting in the Okinawa Trough. In this study, this effect was dealt with by simply increasing the trench retreat rate of the Ryukyu Trench only from 2 Ma to the present as an additional velocity constraint in the numerical simulation. All parameters for the physical properties and subduction history of the PHS plate are tabulated in Tables S1 and S2, respectively.

## Results and discussion

### Thermal structure

As a result of the numerical simulations, when *S* in Eq. (1) was at a minimum with a value of 30.2 ${\text{mW/m}}^{{2}}$(Fig. S3), we obtained optimal values for the retreat rate of the Ryukyu Trench, spreading rate of the Okinawa Trough, and initiation age of trench retreat: 20 mm/year, 15 mm/year, and 7 Ma, respectively. This model is hereafter referred to as a best-fit model.

**Figure 3:**
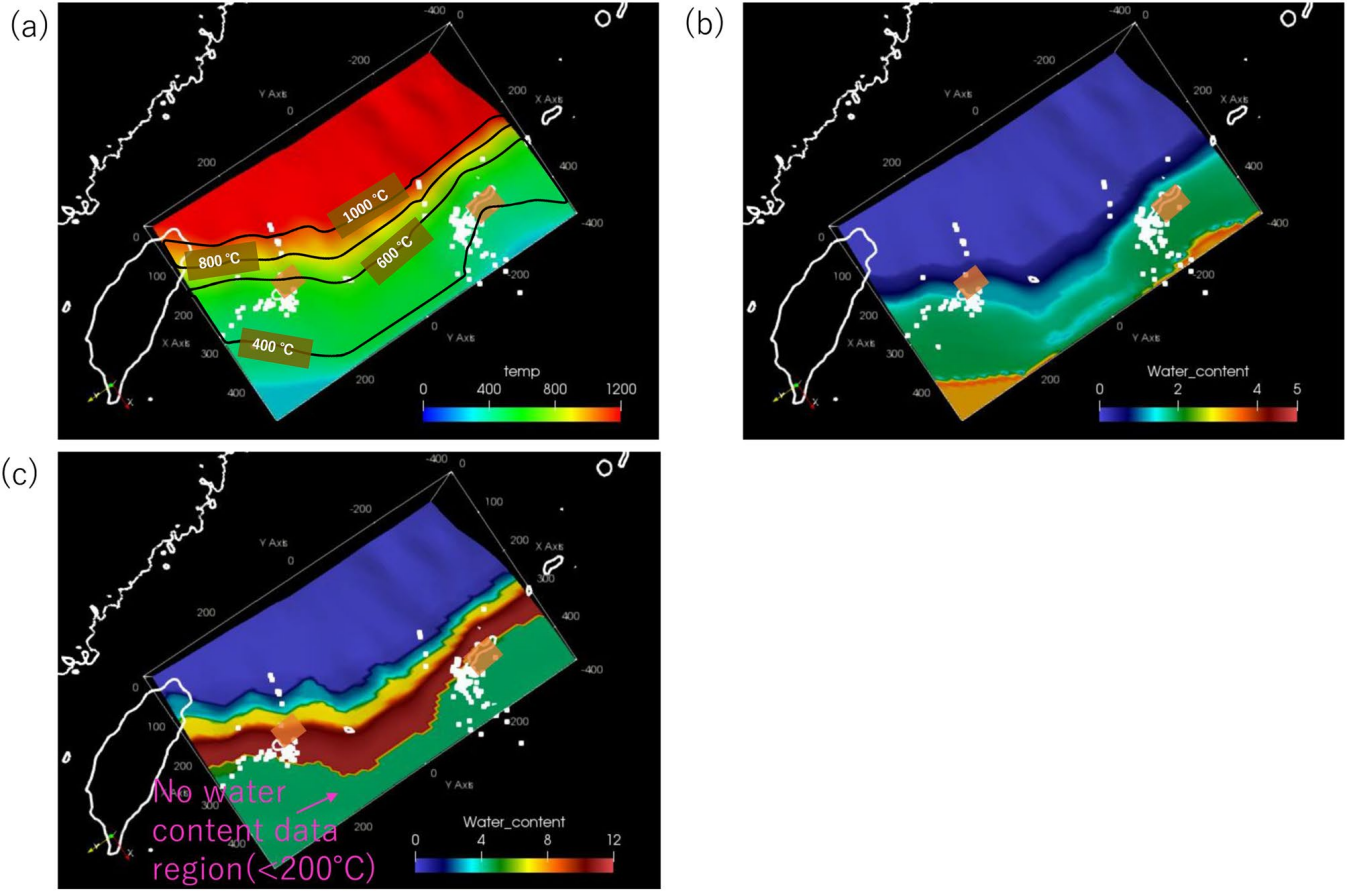
(**a**) Calculated temperature (°C) at the upper surface of the PHS plate for the best-fit model to the observed heat flow data, as viewed from the top. The orange-shaded rectangular areas denote the fault planes of S-SSEs^2^ during the period from 1 January 1997 to 10 November 2013. The white-solid squares denote epicentres of LFEs^3^ during the period from 1 April 2004 to 31 December 2016. The black solid lines denote isothermal contours on the upper surface of the PHS plate. (**b**) Estimated water-content (wt%) distribution obtained using the phase diagram of the hydrous MORB at the surface of the PHS plate for the best-fit model. The others are the same as in (**a**). (**c**) Estimated water-content (wt%) distribution obtained using the phase diagram of hydrous ultramafic rock at a depth of 5 km above the surface of the PHS plate for the best-fit model. The others are the same as in (**a**).

**Figure 4:**
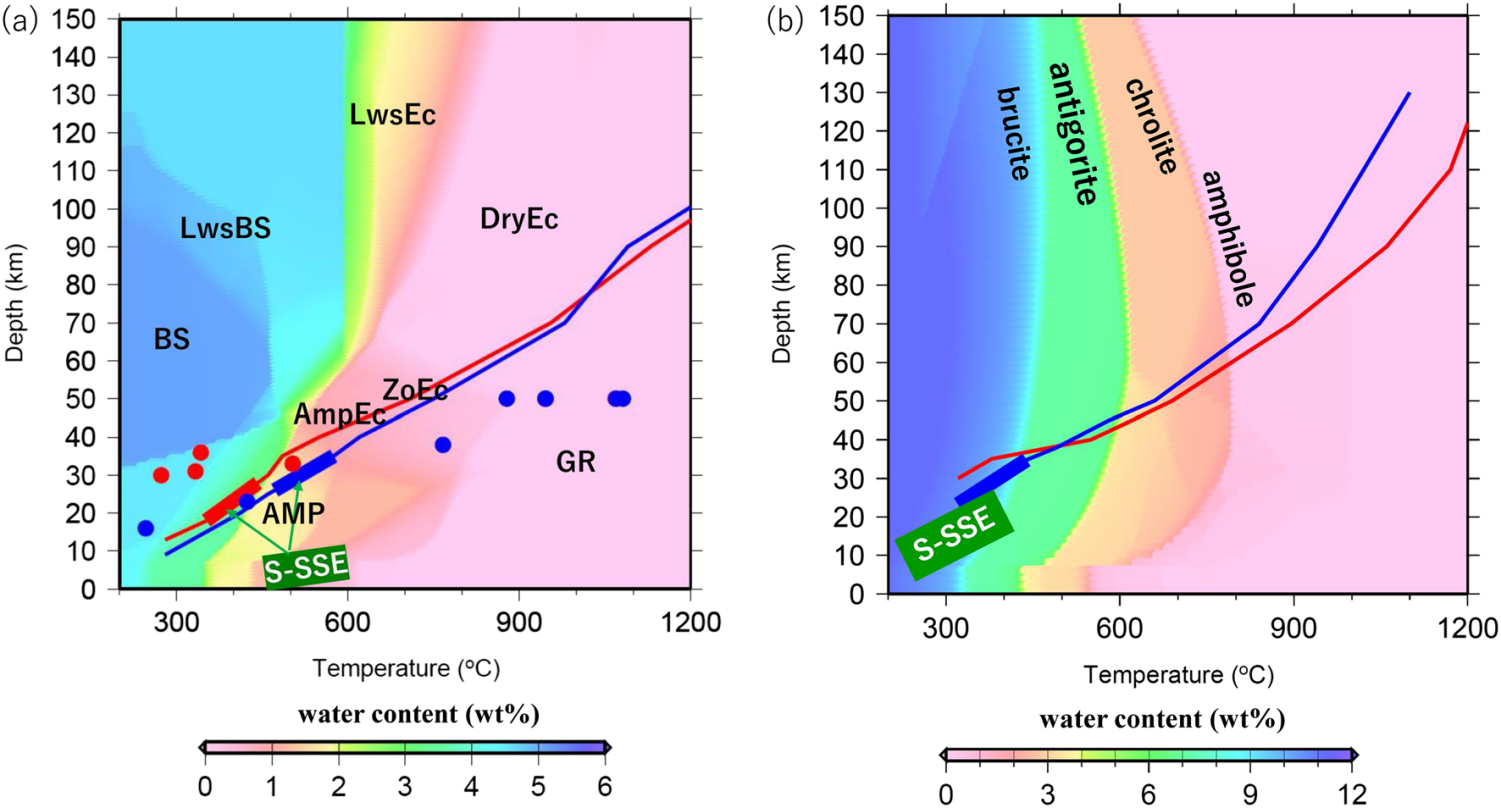
(**a**) Calculated temperature-depth paths in the phase diagram of the hydrous MORB^27^. The red- and blue-solid lines denote the temperature-depth paths at the surface of the oceanic crust of the PHS plate along profiles A and B (Fig. 1), respectively. The thick red and blue lines on the temperature-depth paths denote the temperature-depth ranges of the occurrence of S-SSEs along profiles A and B, respectively. BS, LwsBS, AMP, AmpEC, ZoEC, LwsEc, DryEc, and GR represent the phases of blueschist, lawsonite blueschist, amphibolite, amphibole eclogite, zoisite eclogite, lawsonite eclogite, dry eclogite, and granulite, respectively. (**b**) Calculated temperature-depth paths on the phase diagram of hydrous ultramafic rock^27^. The red- and blue-solid lines denote the temperature-depth paths passing through a depth of 5 km above the surface of the PHS plate along profiles A and B (Fig. 1), respectively. The others are the same as in (**a**).

In the best-fit model, the interplate temperature for the occurrence of S-SSEs and LFEs beneath Okinawa Island ranges from 350 to 450 °C (Fig. 3a). This temperature range for the occurrence of S-SSEs is considered to correspond to the temperature at which the slip mode changes from unstable sliding to stable sliding^8^. On the other hand, temperatures beneath the Yaeyama Islands range from 500 to 600 °C (Fig. 3a), and this range is not very consistent with the above-mentioned transition zone for the slip mode. However, it is not possible to discuss in detail how significant this temperature difference is, taking account of stress field which is not calculated in our kinematic subduction model. The temperature-depth gradients along Okinawa Island (Fig. 4b) are higher than those along the Yaeyama Islands because of the higher dip angle at depths greater than 40 km. Incidentally, the interplate temperature and the mantle wedge temperature, which is taken 5 km above the plate interface, decrease by at most 200 °C when incorporating the effect of trench retreat and the recent spreading rate of the Okinawa Trough into the model; these changes are due to the development of lateral flow in the mantle wedge. The influence of lateral flow in the mantle wedge tends to be stronger after the initiation of spreading of the Okinawa Trough at 2 Ma.

Additionally, the low-temperature region near Okinawa Island in Fig. 3a results from the older seafloor within the model region (Fig. S2(d)). On the other hand, the subduction velocity around the Yaeyama Islands is faster than that around Okinawa Island. Thus, the cold slab can easily reach greater depths around the Yaeyama Islands. The former and latter effects have a trade-off factor, although the former effect has a greater influence on the thermal regime, considering the lower temperature-depth gradient around Okinawa Island (Fig. 4a). However, lateral mantle flow in the along-arc direction associated with oblique subduction could also be a factor causing the difference in interplate temperature at the same depth between the two regions. However, such flow almost ceased after the PHS plate changed its motion at 3 Ma because the PHS plate was subducting nearly parallel to the across-arc direction. From these considerations, explaining why S-SSEs occur at a deeper location beneath the Yaeyama Islands in terms of the thermal state only is difficult.

### Water content distributions

We also calculated the water content distributions near the plate interface using the calculated thermal structure and phase diagrams for hydrous minerals in the oceanic crust and the mantle wedge, which were obtained from the Perple_X program^27^ (Fig. 4a,b). We assumed a sharp dehydration transition when a certain hydrous mineral within the oceanic crust or the mantle wedge transformed into another higher P–T mineral. The results show that the shallower region where S-SSEs and LFEs occur beneath Okinawa Island is adjacent to the deeper region where the phase transformation from blueschist to amphibolite within the oceanic crust takes place (Figs. 3b and 4a). In contrast, the phase transformation from amphibolite to amphibole eclogite within the oceanic crust takes place close to the S-SSE fault plane around the Yaeyama Islands (Figs. 3b and 4a). We also investigated a temperature-depth path at a depth of 5 km above the slab surface (Fig. 4b). Consequently, phase transformations from brucite to antigorite and antigorite to chlorite in ultramafic rocks were identified beneath the Yaeyama Islands (Figs. 3c and 4b). The phase transformation from brucite to antigorite was identified at depths 5–10 km deeper than the S-SSE occurrence region along profile A, passing through Okinawa Island (Figs. 3c and 4b).

Comparing our results with the ${\text{V}}_{{\text{p}}} {\text{/V}}_{{\text{s}}}$ ratio estimated from seismic tomography^28^ (Fig. S4), we found that the region with a ${\text{V}}_{{\text{p}}} {\text{/V}}_{{\text{s}}}$ ratio appropriate for the occurrence of slow earthquakes (1.75 < ${\text{V}}_{{\text{p}}} {\text{/V}}_{{\text{s}}}$ < 1.85^29^) corresponded well with the region where the phase transformation from brucite to antigorite within the slab mantle was identified in our results. In this study, we did not introduce a particle trace system for slab-derived fluid, but some preceding studies performed numerical simulations and showed that wet, slab-derived water or slab melt upwelled, passing through the mantle wedge^30–31^. In summary, almost the same amount of slab-derived water was supplied near the S-SSE occurrence regions beneath both Okinawa Island and the Yaeyama Islands and was associated with phase transformations in the oceanic crust despite a depth difference in the occurrence of S-SSEs. The phase transformations within the mantle wedge along Okinawa Island were shallower than those along the Yaeyama Islands (Fig. 4b) because the steeper dip angle along Okinawa Island may have led to mantle upwelling. However, phase transformations within the mantle wedge occurred in the region slightly deeper than the S-SSE fault planes.

In our future work, there are some challenges for improving our model to make it more realistic. For example, we need to trace the particle motion of the derived fluid, which would change the pore fluid pressure on the plate interface and the spatiotemporal frictional properties^9–10^. In addition, it is important to consider the changes in the physical properties of mantle rocks associated with their mineral metamorphism, such as serpentinization^9,14^. We also need to introduce the effects of chemical reactions associated with equilibrium or non-equilibrium phase transformations considering the latent heat effect on solid–solid reactions in crustal and mantle rocks. As pressure and temperature conditions change, the physical properties of crustal and mantle rocks are affected by structural changes in the crystal lattices of minerals and the absorption or release of latent heat associated with their phase transitions^32^.

## Conclusions

In this study, we performed three-dimensional thermomechanical subduction modelling of the Ryukyu subduction zone to investigate the cause of depth differences in S-SSEs and LFEs in terms of temperature field and dehydration processes. The results of this study can be summarised as follows:

1. The most suitable parameter values to explain the observed heat flow data for the retreat rate of the Ryukyu Trench, spreading rate of the Okinawa Trough, and initiation age of trench retreat are estimated to be 20 mm/year, 15 mm/year, and 7 Ma, respectively.
2. The interplate temperatures where S-SSEs take place range from 350 to 450 °C and 500 to 600 °C beneath Okinawa Island and the Yaeyama Islands, respectively. The latter range is approximately 150 °C higher than the former. Because the effect of older seafloor around Okinawa Island has a larger influence on the thermal regime than the increasing subduction velocity towards the southwest, the interplate temperature around Okinawa Island is more likely to become colder than that around the Yaeyama Islands.
3. In the S-SSE occurrence region, phase transformations from blueschist to amphibolite and from amphibolite to amphibole eclogite within the oceanic crust are inferred near Okinawa Island and the Yaeyama Islands, respectively. On the other hand, the phase transformations from brucite to antigorite and from antigorite to chlorite within the mantle wedge are identified only beneath the Yaeyama Islands.
4. The estimated amount of slab-derived water coming from the oceanic crust becomes almost the same beneath both Okinawa Island and the Yaeyama Islands near the S-SSE occurrence regions despite the difference in temperature-depth conditions between them.

## Methods

In this study, we used an anelastic liquid approximation. For the governing equations, we used mass conservation, momentum, and energy equations to calculate the temperature and mantle flow velocity fields. The mass conservation equation can be written as

2 $$ \nabla \cdot \left[ {\rho_{s} \left( z \right){\mathbf{v}}} \right] = 0 $$

where $\rho_{s} \left( z \right)$ and ${\mathbf{v}}$ are the fluid density and flow velocity vectors, respectively. The subscripts denote the adiabatic state. Following Ji et al.^33^, the momentum equation can be written as follows,

3 $$ - \frac{\partial P}{{\partial x_{i} }} + \frac{{\partial \tau_{ij} }}{{\partial x_{j} }} + Ra\alpha \left( {T - T_{s} } \right)\delta_{i3} = 0 $$

where $P$ is the pressure deviation from the hydrostatic pressure, $\tau_{ij}$ is the stress tensor, $R_{a}$ is the Rayleigh number, $\alpha$ is the thermal expansivity, $T$ is the temperature, and $\delta_{ij}$ is Kronecker’s delta. $T$ can be written as

4 $$ \frac{{dT_{s} }}{{dx_{3} }} = \frac{g\alpha D}{{C_{p} }}T_{s} $$

where $g$ is the acceleration of gravity, $D$ is the thickness of the model, and $C_{p}$ is the specific heat at constant pressure. The energy equation is expressed as

5 $$ \rho C_{p} \left( {\frac{\partial T}{{\partial t}} + {\mathbf{v}} \cdot \nabla T} \right) = k\nabla^{2} T + \eta \left( {\nabla v} \right)^{2} + \rho g\alpha Tv + H_{r} \rho + Q_{f} $$

where $t$ is the time, $k$ is the thermal conductivity, $v$ is a vertical component of the flow velocity, $H_{r}$ is the heat generation by a radioactive element, and $Q_{f}$ is the frictional heating on the plate interface. The density $\rho$ is dependent only on temperature.

Following Burkett and Billen^34^, we use viscosity, which is represented by a composite of diffusion creep and dislocation creep:

6 $$ \eta = \frac{{\eta_{diff} \eta_{disl} }}{{\eta_{diff} + \eta_{disl} }} $$

with

7 $$ \eta_{diff,disl} = \left( {\frac{{d^{p} }}{{AC_{OH}^{r} }}} \right)^{\frac{1}{n}} \dot{\varepsilon }^{{\frac{1 - n}{n}}} \exp \left( {\frac{{E + P_{lc} V}}{nRT}} \right) $$

where $\eta_{diff}$ and $\eta_{disl}$ are the viscosities of diffusion and dislocation creeps, respectively, $d$ is the grain size, $p$ is the grain index, $A$ is a coefficient, $C_{OH}$ is the water content, $r$ is the water content index, $n$ is the stress index, $\dot{\varepsilon }$ is the second invariant of the strain rate tensor, $E$ is the activation energy, $P_{lc}$ is the lithostatic pressure defined by the depth gradient of compressibility, $V$ is the activation volume, and $R$ is the gas constant.

The frictional heating $Q_{f}$^35^ can be written as

8 $$ Q_{f} = Min\left( {\tau_{b} ,\tau_{d} } \right)\frac{{v_{t} }}{w} $$

where $\tau_{b}$ and $\tau_{d}$ are the shear stresses at the plate boundary in the brittle and ductile regimes, respectively, $v_{t}$ is the subducting velocity, and $w$ is the thickness of the plate boundary deformation zone. The shear stress $\tau_{b}$ in the brittle regime^36^ can be written as

9 $$ \tau_{b} = 0.85\sigma_{n} \left( {1 - \lambda } \right)\left( {\sigma_{n} \left( {1 - \lambda } \right) \le 200MPa} \right) $$

10 $$ \tau_{b} = 50 + 0.6\sigma_{n} \left( {1 - \lambda } \right)\left( {\sigma_{n} \left( {1 - \lambda } \right) \ge 200MPa} \right) $$

where $\sigma_{n}$ is the normal stress and $\lambda$ is the pore pressure ratio. In the best-fit model (Figs. 3 and 4), the pore pressure ratio $\lambda$ is estimated to be 0.995. The shear stress in the ductile regime^37^ can be written as

11 $$ \tau_{d} = \frac{1}{2}A^{{ - \frac{1}{n}}} \dot{\zeta }^{\frac{1}{n}} \exp \left( {\frac{{E^{\prime}}}{nRT}} \right) $$

where $A$ is a coefficient, $\dot{\zeta }$ is the shear strain rate and $E^{\prime}$ is the activation energy.

By solving Eqs. (2), (3), and (5) as a coupled problem, we can obtain unknown parameters ${\mathbf{v}}$, $P$, and $T$ at each time step.

For the boundary condition for the mantle flow velocity, we imposed permeable conditions at the + x, −x, + y, −y, and + z planes (Fig. 2). When trench retreat was introduced into the model, the model surface was divided into two parts bounded by the time-dependent moving trench axis. On the oceanic-plate side, the oceanward retreat velocity was given on the model surface to realize trench retreat. On the other hand, on the continental-plate side, a flow velocity of (0, 0, 0) was given. As a result, a gap arises between the edge of the continental plate and the trench axis moving away with time. Therefore, we considered a growing accretionary prism with a triangular prism shape increasing with time during the trench-retreat period. The growth rate of the accretionary prism is set to be equal to the trench retreat rate to compensate for the accretionary-prism materials between the trench axis and the continental plate.

For the boundary condition for the temperature fields, we assigned adiabatic conditions at the − x, + y, − y, and + z planes. A fixed temperature of 0 °C was imposed at the − z plane, and the temperature *T*(*z*, *t*) at the + x plane was determined by the plate cooling model^38^:

12 $$ T\left( {z,t} \right) = T_{0} + \left( {T_{1} - T_{0} } \right)\left[ {\frac{z}{{d_{l} }} + \frac{2}{\pi }\sum\limits_{n = 1}^{\infty } {\frac{1}{n}\exp \left( { - \frac{{\kappa n^{2} \pi^{2} t}}{{d_{l}^{2} }}} \right)\sin \left( {\frac{n\pi z}{{d_{l} }}} \right)} } \right] $$

where $T_{0}$ and $T_{1}$ are the surface and basal temperatures of the lithosphere, respectively, $z$ is the depth, $d_{l}$ is the depth at which the mantle temperature reaches $T_{1}$, $n$ is an index, $\kappa$ is the thermal diffusivity, and $t$ is the age of the PHS plate at the Ryukyu Trench. We used the RT1 model^39^, in which values of $T_{1}$ and $d_{l}$ are given as 1402 °C and 134.9 km, respectively.

The initial condition in the model domain is a steady state without mantle flows. Following Yoshioka and Sanshadokoro (2002)^40^, the temperature distribution for the half-space cooling model can be expressed as

13 $$ T = T_{0} erf\left( {\frac{z}{{2\sqrt {\frac{{kt^{\prime}}}{{\rho C_{p} }}} }}} \right) $$

and

14 $$ T = T_{0} erf\left( {\frac{z}{{2\sqrt {\frac{{kt^{\prime}}}{{\rho C_{p} }}} }}} \right)\exp \left[ {\frac{g\alpha }{{C_{p} }}\left( {z - z_{0} } \right)} \right] $$

where $T_{0}$ is a potential temperature, $t^{\prime}$ is the age of the continental plate, and $z_{0}$ is the depth below which the effect of adiabatic compression is considered. When $z \ge z_{0}$, we used Eq. (14) instead of Eq. (13), taking into account adiabatic compression.

## Supplementary Information

Supplementary Information.
