# Supplementary material for: 3-D thermal regime and dehydration processes around the regions of slow earthquakes along the Ryukyu Trench
Source: Sci Rep. 2021 May 27;11:11251. doi: 10.1038/s41598-021-90199-2 (PMC8160006; doi:10.1038/s41598-021-90199-2)
Supplement: Supplementary file 1 — Supplementary Information. [file 41598_2021_90199_MOESM1_ESM.docx]

Supporting information for

“3-D thermal regime and dehydration processes around the regions of slow earthquakes along the Ryukyu Trench

”

Nobuaki Suenaga^1,^*, Shoichi Yoshioka^1,2^, and Yingfeng Ji^3,4^

^1^ Research Center for Urban Safety and Security, Kobe University, Rokkodai-cho 1-1, Nada ward, Kobe 657-8501, Japan

^2^ Department of Planetology, Graduate School of Science, Kobe University, Rokkodai-cho 1-1, Nada ward, Kobe 657-8501, Japan

^3^ State Key Laboratory of Tibetan Plateau Earth System Science (LATPES), Institute of Tibetan Plateau Research, Chinese Academy of Sciences, Beijing 100101, China

*corresponding author

TEL: +81-90-8415-1989

FAX: +81-78-803-6598

e-mail: [n.suenaga@people.kobe-u.ac.jp](mailto:n.suenaga@people.kobe-u.ac.jp)

Contents

Table S1-S2

Figures and figure captions S1-S4

References

**Table S1 Physical properties for temperature and flow field.**

| parameters for calculating temperature field | | |
| --- | --- | --- |
| specific heat at constant pressure $C_{p}$ $\left(\text{Jkg}^{-1}\text{K}^{-1}\right)$   | 　　　 　　  | |
| thermal expansivity $\alpha$ $\left(\text{K}^{-1}\right)$   |   | |
| potential temperature $T_{0}$ $\left({}^{\circ}\text{C}\right)$   | 1350  | |
| standard density of mantle $\rho_{0}$ $\left(\text{kgm}^{-3}\right)$   |   | |
| Parameters for calculating viscosity  | diffusion  creep | dislocation  creep |
| stress index $n$  | 1.0 | 3.5 |
| coefficient $A$ $\left(\text{s}^{-1}\text{Pa}^{-\text{n}}\text{μm}^{\text{p}}\text{C}_{\text{OH}}^{-\text{r}}\right)$   | 1.0 | $9.5\times10^{-20}$ |
| activation energy $E$ $\left(\text{kJmol}^{-1}\right)$   | 335 | 480 |
| activation volume $V$ $\left(\text{m}^{3}\text{mol}^{-1}\right)$   | $4.0\times10^{-6}$ | $11\times10^{-6}$ |
| grain size $d$ $\left(\text{μm}\right)$   | 10000 | ― |
| grain index $p$  | 3.0 | ― |
| water content $C_{OH}$ $\left(\text{ppm}\right)$  | 1000 | 1000 |
| water content index $r$  | 1.0 | 1.2 |

(1) Yoshioka and Sanshadokoro (2002); (2) Honda (1997); (3) Takenaka et al. (1999); (4) Burkett and Billen (2010)

**Table S2 Tectonic setting.**

| Period（Ma） | Migration rate of the PHS  plate in the along-arc direction  [±y direction]  （cm/yr） | Retreat rate of the PHS  plate in the across-arc direction  [±x direction]  （cm/yr） | Subduction history |
| --- | --- | --- | --- |
| 15-7 | -2.2 | 0.0 | ・Northeastward migration of the PHS plate  ・Northeastward migration of the PHS plate  ・Retreat of the Ryukyu Trench |
| 7-3 | -2.2 | 2.0 |  |
| 3-2 | 0.2 | 2.0 | ・Northwestward migration of the PHS plate  ・Retreat of the Ryukyu Trench  ・Northwestward migration of the PHS plate  ・Retreat of the Ryukyu Trench  ・Spreading of the Okinawa Trough |
| 2-0 | 0.2 | 3.5 |  |

**
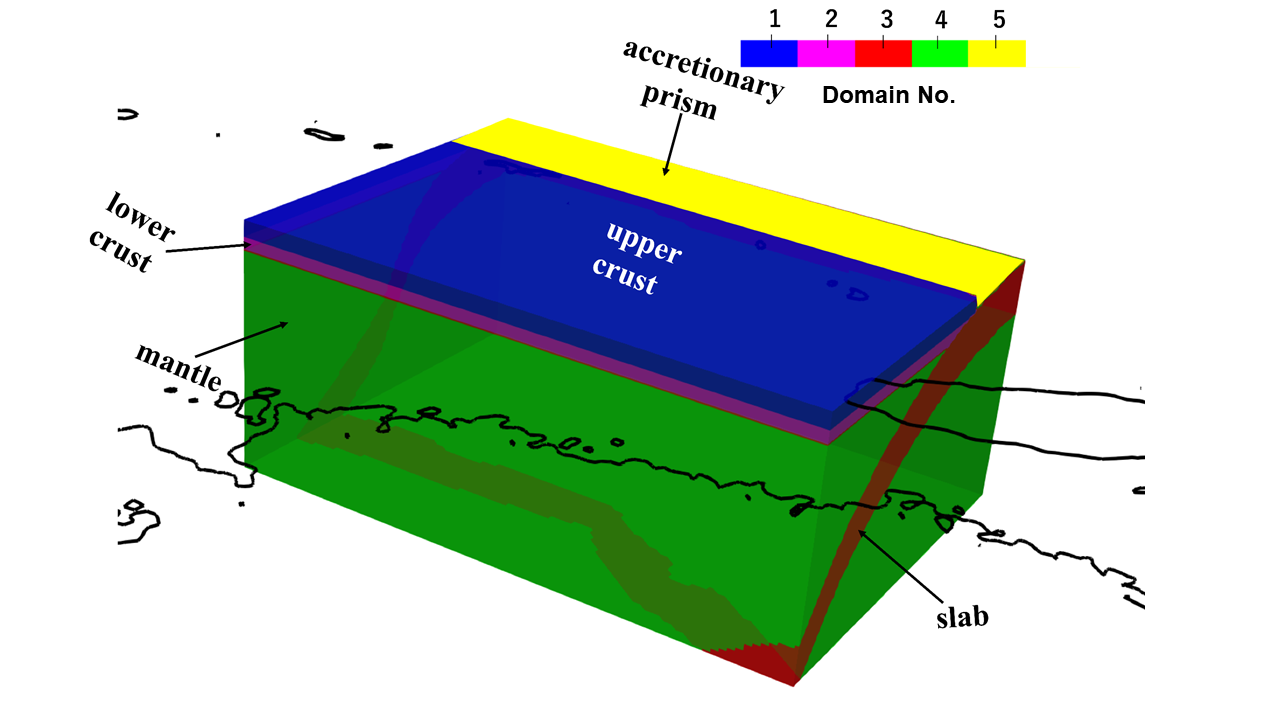
**

**Figure S1**

Figure S1 **Bird’s eye view** of the three-dimensional thermomechanical subduction model consisting of five portions. Domains 1, 2, 3, 4, and 5 denote the upper crust, lower crust, slab, mantle, and accretionary prism, respectively.

**Figure S2**


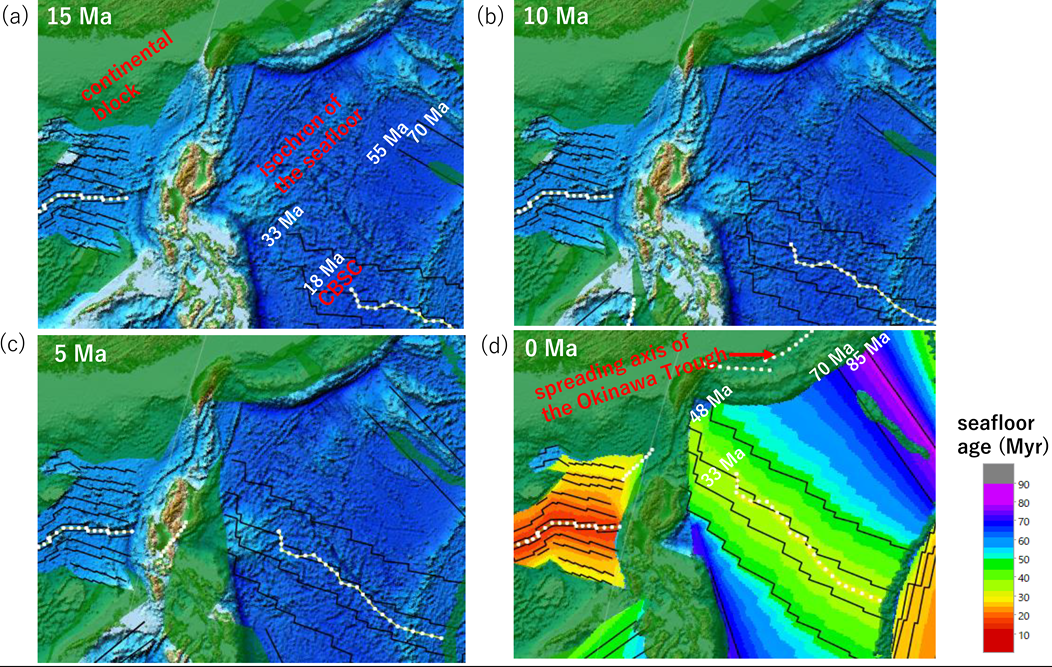


Figure S2 Snapshots of the plate-rotation model. The Black solid lines denote the isochrons on the seafloor at the labelled ages. The green shaded regions denote the continental blocks. The white-dotted lines denote the Central Basin Spreading Centre (CBSC) and the spreading axis of the Okinawa Trough. Color contours in (d) denotes the current oceanic ages in and around the Ryukyu subduction zone. (a) 15 Ma. (b) 10 Ma. (c) 5 Ma. (d) 0 Ma.

**Figure S3**


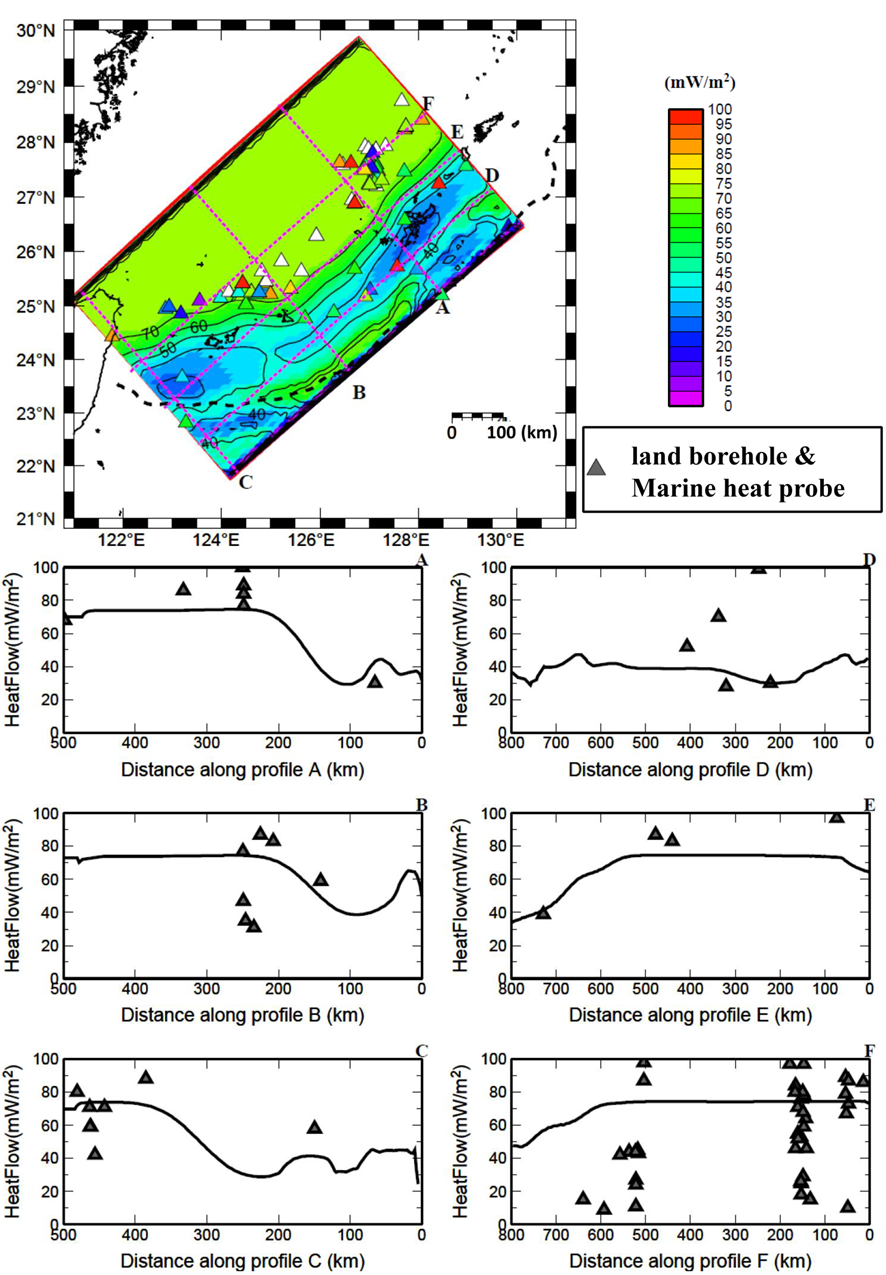


Figure S3 (top panel) Comparison between the observed and calculated surface heat flow distributions for the best-fit model. The colored-solid triangles and contours denote observed and calculated heat flow distributions, respectively. (Lower 6 panels) Observed (black-solid triangles) and calculated (black lines) heat flows along the six profiles A to F in the top panel. The observed heat flow data are taken within a one-sided width of 30 km along each profile.


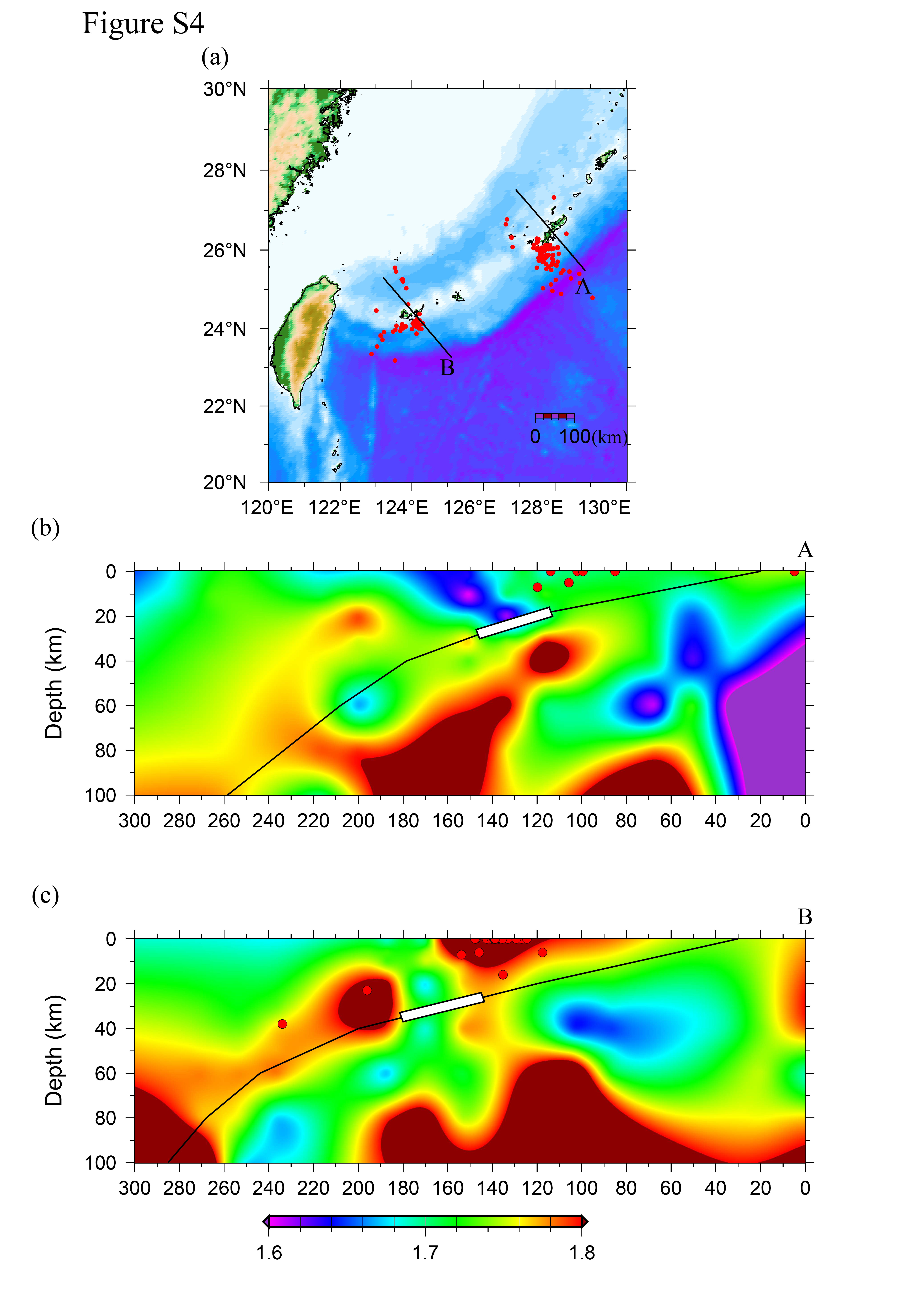


**Figure S4**

Figure S4 Spatial distributions of $\text{V}_{\text{p}}\text{/V}_{\text{S}}$ ratios. (a) Locations of profiles A and B (black lines) along which the $\text{V}_{\text{p}}\text{/V}_{\text{S}}$ ratio distributions are shown in (b) and (c), respectively, and the LFE distributions (red dots). (b) Vertical cross section of the $\text{V}_{\text{p}}\text{/V}_{\text{S}}$ ratio distribution along profile A in (a). The black-solid line denotes the upper surface of the PHS plate. The thick white line surrounded by a black frame on the upper surface of the PHS plate denotes the locations where S-SSE occur along profile A. The red dots denote the hypocentre distributions of LFEs within a one-sided width of 20 km along profile A during the period from 1 October 1997 to 31 December 2015. (c) Same as (b) except for profile B.

**References**

SI1. Honda, S. Mantle dymanics II—mechanics, in Iwanami-koza. Earth and Planetary Science 10 Dynamics of the Earth’s interior, Iwanami-Shoten, Tokyo. pp. 73–121 (in Japanese) (1997).

SI2. Takenaka, S., Sanshadokoro, H. & Yoshioka, S. Velocity anomalies and spatial distributions of physical properties in horizontally lying slabs beneath the Northwestern Pacific region. Phys. Earth Planet. Int. 112, 137-157 (1997).

SI3. Müller, R. D., Seton, M., Zahirovic, S., Williams, S.E., Matthews, K.J., Wright, N.M., Shephard, G.E., Maloney, K.T., Barnett-Moore, N., Hosseinpour, M., Bower, D.J., Cannon, J. Ocean basin evolution and global-scale plate reorganization events since Pangea breakup. Annual Reviews of Earth and Planetary Sciences, 44, 107-138 (2016).
